# Lung Adenocarcinoma With Extensive Vascular Invasion and Retinal Metastasis: A Case Report

**DOI:** 10.1002/rcr2.70170

**Published:** 2025-06-05

**Authors:** Bassem Alhariri, Arwa Elfatih, Osman Osama Elhassan, Mohamed Elfatih, Aya Mohamed Kheir, Razan Elkhatib

**Affiliations:** ^1^ Department of Medicine Hamad Medical Corporation Doha Qatar; ^2^ College of Medicine Qatar University Doha Qatar; ^3^ College of Medicine Weill Cornell Medicine—Qatar Doha Qatar; ^4^ Medical Education Department Hamad Medical Corporation Doha Qatar

**Keywords:** lung adenocarcinoma, non‐small cell lung cancer, retinal metastasis, targeted therapy, vascular invasion

## Abstract

A 34‐year‐old Ghanaian male with no past medical history presented to our hospital with dyspnoea, left‐sided chest pain, and left‐eye visual disturbance. Imaging revealed a large mediastinal mass with vascular invasion, left pleural and skeletal metastases. A lung biopsy confirmed lung adenocarcinoma (LUAD) with mixed solid and lepidic growth patterns, showing 80% PD‐L1 expression. Positron emission tomography (PET) identified a highly active lesion in the left eye, suggestive of retinal metastasis. Ophthalmologic evaluation confirmed retinal involvement. Given ROS1 positivity, Crizotinib was initially prescribed but switched to Lorlatinib due to concerns regarding retinal involvement. The patient demonstrated rapid clinical improvement, including notable visual recovery, with stable performance status after 1 month. This case underscores LUAD's potential for atypical metastasis, such as to the retina, necessitating comprehensive assessment and personalised management strategies. The shift from Crizotinib to Lorlatinib highlights the importance of targeted therapy in managing advanced NSCLC with rare metastatic involvement.

## Graphical abstract

This case report describes a unique presentation of lung adenocarcinoma with extensive vascular invasion and retinal metastasis, emphasising the role of molecular profiling and targeted therapy in managing advanced disease.

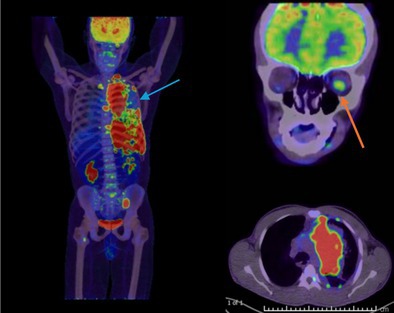

## 1 Introduction

Lung cancer remains the leading cause of cancer‐related deaths globally, with an estimated 1.6 million deaths annually. Lung adenocarcinoma (LUAD) and lung squamous cell carcinoma (LUSC) are the two most prevalent histological subtypes of NSCLC, accounting for approximately 85% of cases. LUAD, the most common subtype in non‐smokers, is associated with genetic mutations such as EGFR, ALK, and ROS1, which are actionable targets for tyrosine kinase inhibitors (TKIs) [1]. Retinal metastasis from LUAD is exceedingly rare, with fewer than 50 cases reported in the literature. This case report describes a unique presentation of LUAD with extensive vascular invasion and retinal metastasis, emphasising the role of molecular profiling and targeted therapy in managing advanced disease.

## 2 Case Report

### 2.1 Patient History

A 34‐year‐old Ghanaian male presented to the emergency department of Hamad Medical Corporation, Doha, Qatar, in March 2023, with a two‐day history of progressively worsening dyspnoea and left‐sided chest pain, accompanied by blurred vision in the left eye. He reported a two‐month history of productive cough, intermittent fever, night sweats, and unintentional weight loss of 5 kg.

### 2.2 Imaging and Diagnostic Workup

**FIGURE 1:**
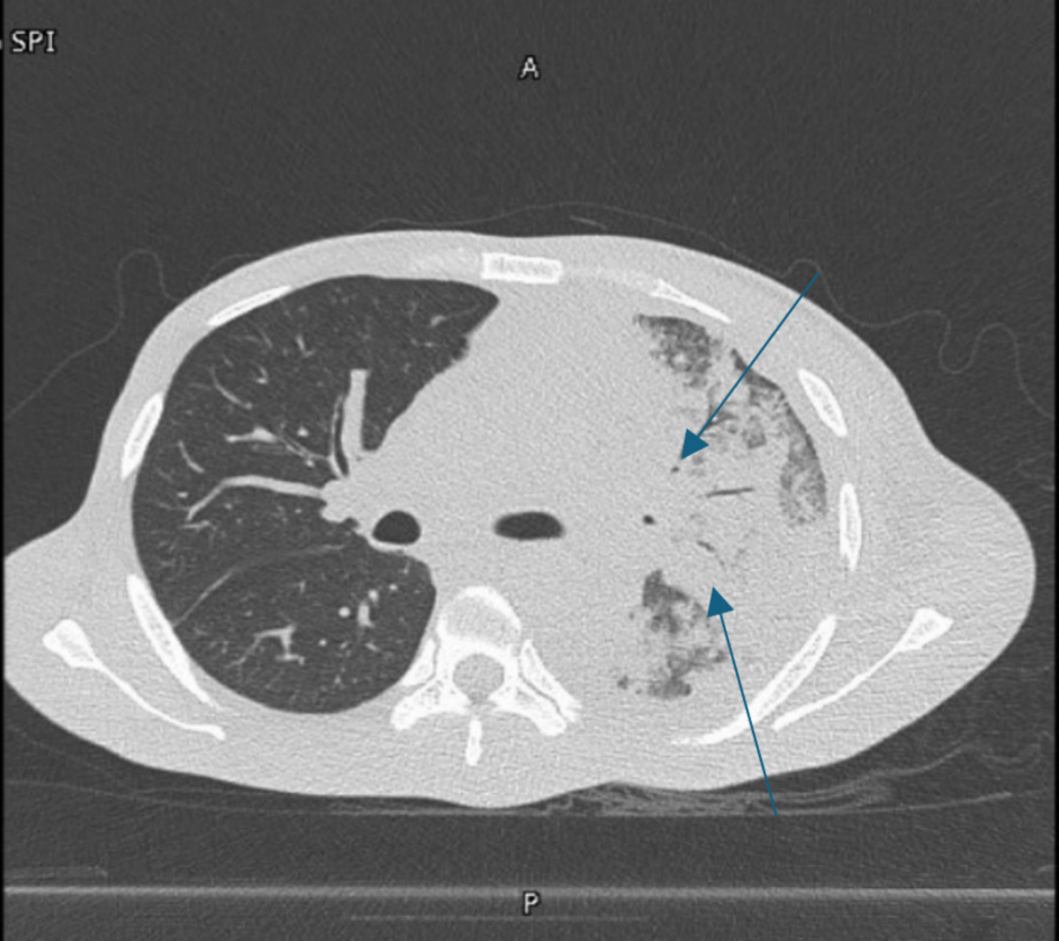
A contrast‐enhanced CT of the thorax showing a large malignant mediastinal mass invading the left brachiocephalic trunk blue arrows.

**FIGURE 2:**
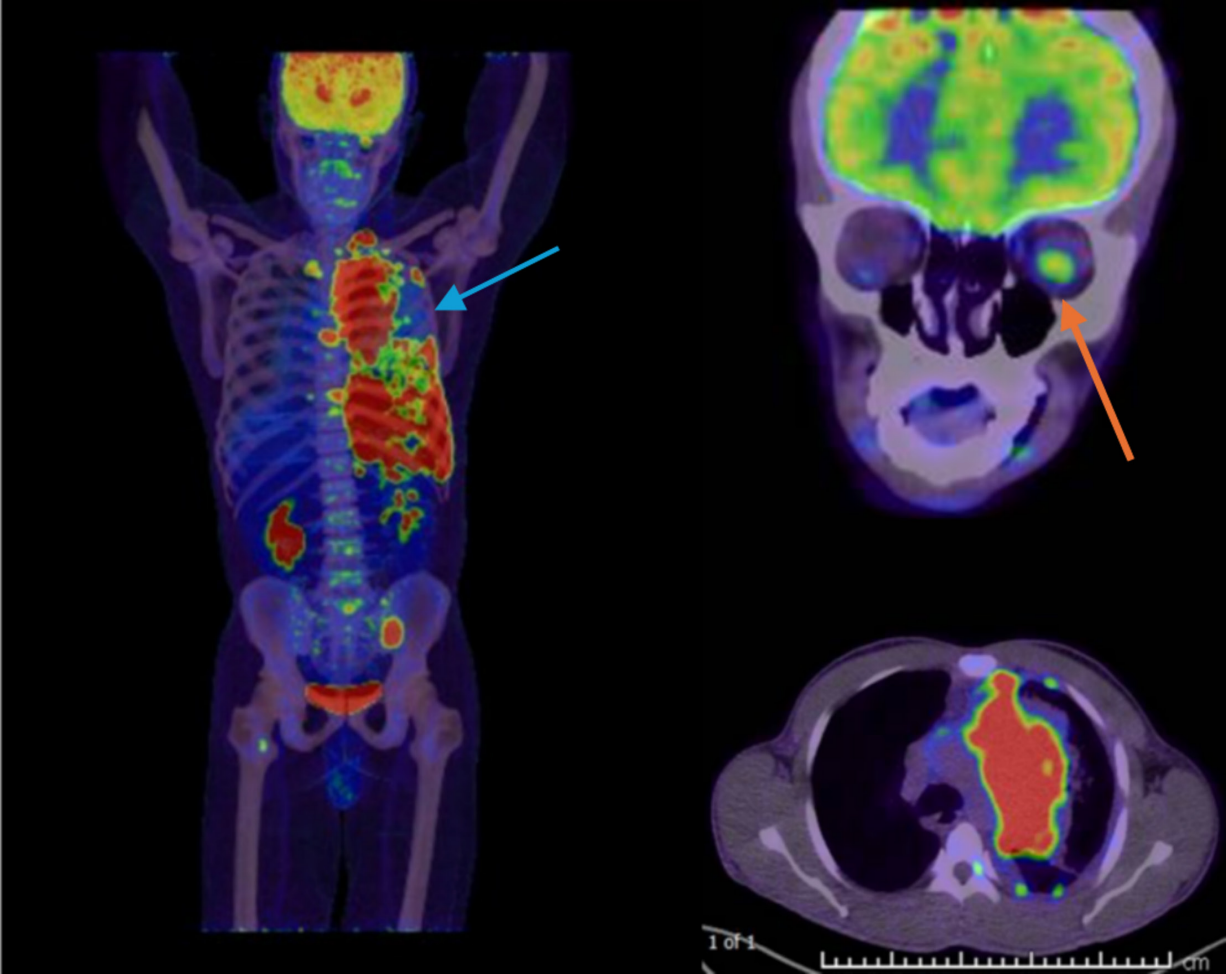
PET Scan showing soft tissue mass in the left hemithorax green arrow, focal intense metabolic activity at the posterior aspect of the left eye red arrow.

A contrast‐enhanced chest computed tomography (CT) scan revealed a large malignant mediastinal mass invading the left brachiocephalic trunk, left internal jugular vein, and left subclavian vein, causing thrombosis (Figure 1, blue arrow). The mass encased the upper lobe branch of the left pulmonary artery and was associated with multiple enlarged left axillary lymph nodes and left pleural metastases. Positron emission tomography‐computed tomography (PET‐CT) with fluorodeoxyglucose identified an 8 cm × 12.5 cm soft tissue mass in the left hemithorax (SUVmax 35.8) and a 1.6 cm left supraclavicular lymph node (SUVmax 17.5). Notably, focal intense metabolic activity was observed at the posterior aspect of the left eye (SUVmax 9.9, Figure 2, red arrow).

### 2.3 Ophthalmologic Evaluation

**FIGURE 3:**
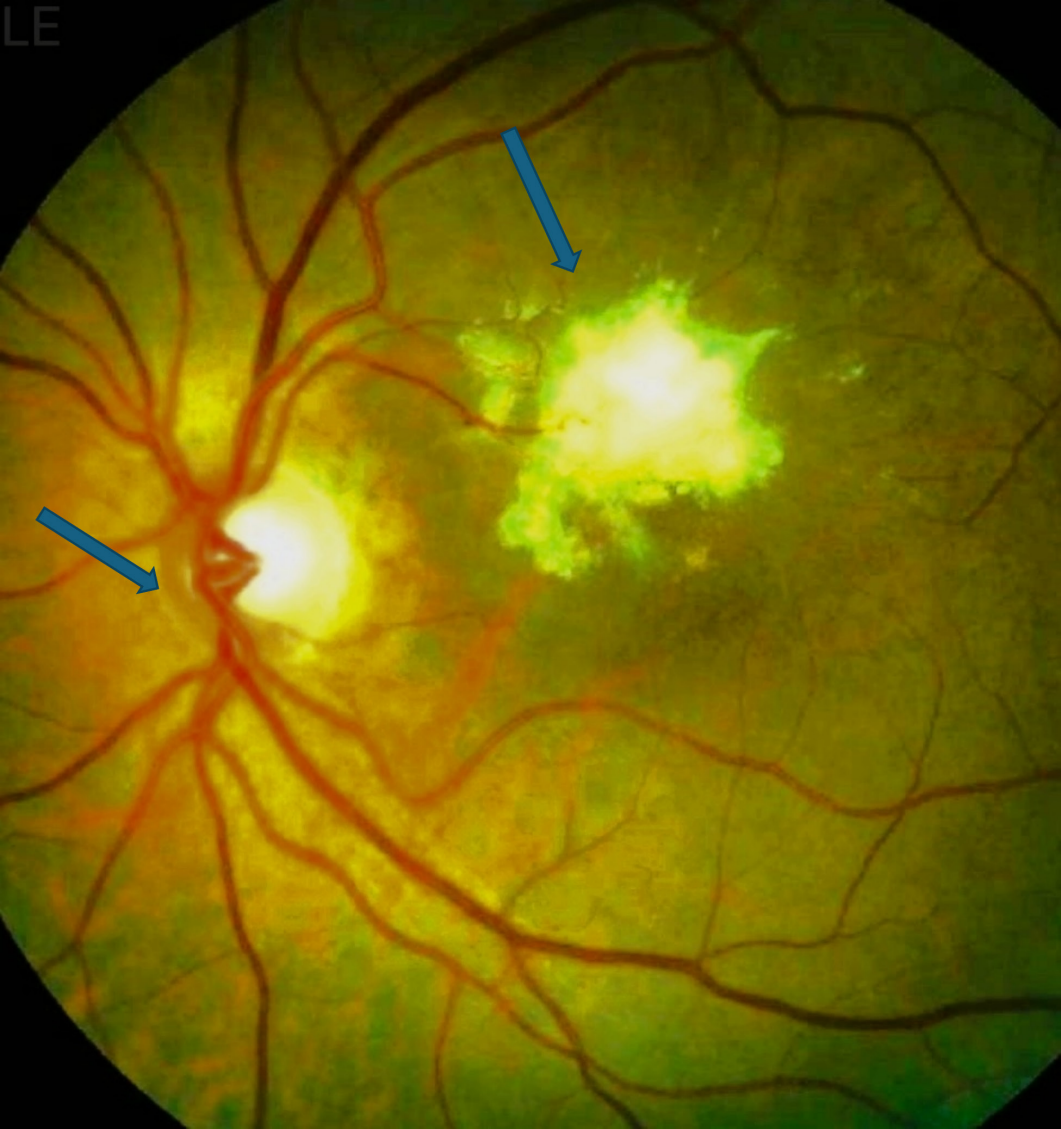
Intraretinal lesion with ill‐defined margins and vitreous seeding in the left eye/fundoscopic photograph of a left eye showing features of retinal metastasis from lung adenocarcinoma ➔ (blow arrows).

Fundoscopic examination revealed a yellow white intraretinal lesion with ill‐defined margins and vitreous seeding in the left eye (Figure 3). Optical coherence tomography (OCT) demonstrated subretinal fluid accumulation and retinal thickening (Table 1).

**TABLE 1:** Ophthalmologic evaluation.

Exam	Right eye	Left eye
Visual acuity	6/6	HM unaided
Eyelids	Normal	Normal
Lacrimal	Patent	Patent
Conjunctiva and sclera	Clear	Quiet
Cornea	Clear	Clear
Anterior chamber (AC)	Quite	Quite
Iris	Normal	Normal
Pupil	RRR	RRR
Lens	Clear	Clear
Ocular motility	Full painless	Full painless
Fundus	Normal macula and disc	**Splinter haemorrhage on the disc and macula nasal choroidal involvement with overlying exudative retinal detachment**

*Note:* Bold text indicates abnormal findings.

Abbreviation: RRR, round, regular, reacting to light.

### 2.4 Pathologic and Molecular Findings

A CT‐guided percutaneous lung biopsy demonstrated pulmonary adenocarcinoma with mixed solid and lepidic growth patterns. Immunohistochemistry (IHC) for ALK (5A4) was negative, while PD‐L1 expression was observed in 80% of tumour cells. Next‐generation sequencing (NGS) identified a ROS1 rearrangement.

### 2.5 Treatment and Clinical Course

**FIGURE 4:**
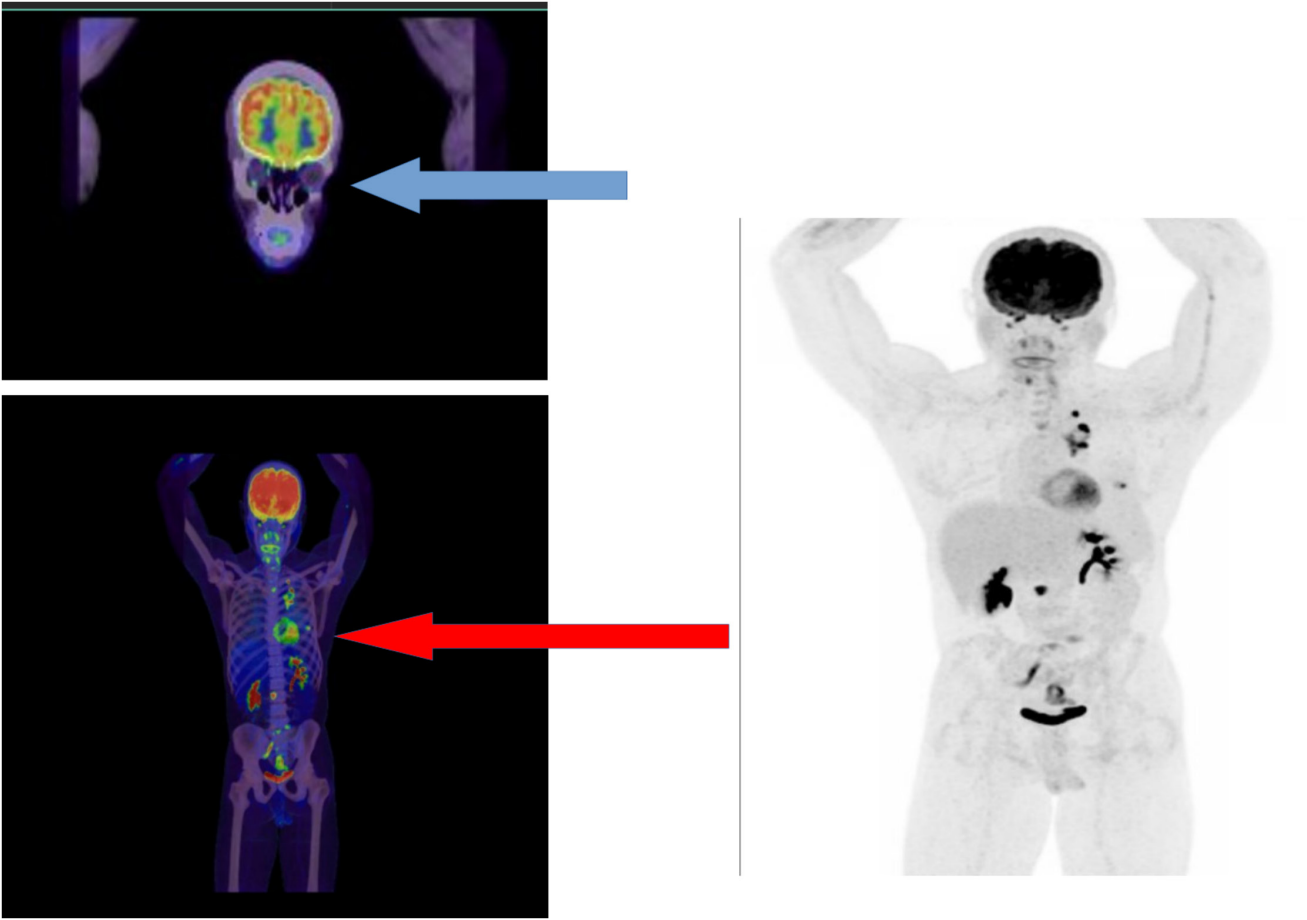
PET scan showing the regression of the hypermetabolic lesions which were identified on prior study. Blue arrow: Complete regression of Intensely hypermetabolic in the left eye posterior aspect nodule. (No longer noted compared to prior PET scan.) Red arrow: Intensely hypermetabolic left pleural nodules are no longer seen and left Para mediastinal mass is markedly regressed.

Initial treatment with Crizotinib 250 mg twice daily was initiated due to ROS1 positivity. However, due to concerns regarding retinal metastasis and limited central nervous system penetration of Crizotinib, therapy was switched to Lorlatinib 100 mg daily. The patient demonstrated rapid clinical improvement, becoming oxygen‐independent within 72 h. At one‐month follow‐up, his visual acuity improved from 20/200 to 20/40, and repeat PET‐CT showed reduced metabolic activity in the left eye (SUVmax 2.1) and mediastinal mass (SUVmax 12.4). Below we attached the PET scan studies after a course of 2 months of chemotherapy as mentioned above (Figure 4).

## 3 Discussion

Lung adenocarcinoma (LUAD) can involve blood vessels, making diagnosis difficult and impacting patient outcomes. Pulmonary artery involvement in LUAD is rare but important to recognise, as it can mimic pulmonary thromboembolism on imaging. This similarity can lead to misdiagnosis without advanced imaging like PET‐CT scans and biopsy. Studies have documented that (LUAD) can cause pulmonary artery filling defects, resembling thromboembolism but actually representing tumour invasion, highlighting the importance of distinguishing between these presentations early in the course of disease [2, 3].

Subclavian artery thrombosis, another unusual (LUAD) presentation, can result from direct tumour invasion or cancer‐related hypercoagulability [4]. These cases emphasise the need for thorough assessment and advanced imaging to differentiate malignancy‐driven vascular involvement from primary thromboembolic events. Recognising these (LUAD) presentations is crucial for timely and appropriate treatment, as misdiagnosis can delay necessary oncologic therapy [5].

Metastatic cancer to the eye is considered rare; it occurs primarily through haematogenous spread, with the choroid being the most affected site due to its rich vascular supply. Incidence in the United States is estimated to be around 20,000 cases yearly. Lung and breast cancers are the most common major causes, making up 21% and 47% of cases, respectively.

Most of these patients have not been seen by an ophthalmologist. It has been argued that it is due to the fact that they have no visual complement due to more serious systemic medical issues. As a result, some have questioned the need for eye screening of all patients with metastatic disease [6].

Retinal metastases are usually unilateral and solitary; however, bilateral and multifocal involvement has also been reported. At the time of diagnosis of retinal metastasis, many patients have metastases at other sites. Alternatively, ocular involvement may be the first finding of metastatic disease, and these patients must be carefully evaluated for the presence of systemic metastases at other sites [1].

Of the 41 cases with carcinoma metastasis to the retina, 15 (36.6%) cases had lung carcinoma, 13 (31.7%) cases had gastrointestinal carcinoma including 1 case of pancreatic carcinoma and 1 case of hepatocholangiocarcinoma, 6 (14.6%) had breast carcinoma, 3 (7.3%) had adenocarcinoma with unknown primary site, 2 (4.9%) had genitourinary tract carcinoma, 1 (2.4%) had uterine carcinoma, and 1 (2.4%) had nasopharyngeal carcinoma. The mean age of all reported cases was 56.1 (range 15–75, SD ± 11.9) years.

The mean survival for all reported cases of retinal metastasis was just 5.7 (range 1–23, SD ± 5.2) months, indicating a dismal survival rate for individuals with carcinoma metastases to the retina [1].

The common presenting symptoms of carcinoma metastases to the retina include ocular discomfort, floaters, and diminished vision. Retinal metastases, however, may be asymptomatic. The most typical presentation of retinal metastases is a localised, intraretinal tumour that is yellow‐white and has ill‐defined boundaries. But occasionally, the tumour's boundaries might be well‐circumscribed. It is possible to see underlying vitreous cells that resemble uveitis. White‐yellow cellular clumps or strands can indicate vitreous involvement, which can occasionally impair retinal vision. Anterior chamber cells and a tumour cell‐formed pseudo hypopyon are occasionally also visible. Glaucoma may develop as a result of tumour cells in the trabecular meshwork obstructing aqueous outflow [1].

In our case, the patient complained of an acute onset of unilateral headache, orbital pain, and severe photophobia. An MRI head with contrast was done, and brain metastasis was ruled out. Then, he was referred to an ophthalmologist, and a visual evaluation was done. The right eye fundus exam showed a Splinter haemorrhage on the disc and macula nasal choroidal involvement with overlying exudative retinal detachment. Eventually, a PET scan confirmed an intense metabolic activity at the posterior aspect of the left eye (SUVmax 9.9), accompanied by soft tissue thickening, going with the suggestion of retinal metastasis.

Carcinoma metastatic to the retina presents a challenging clinical scenario with limited therapeutic options and a dismal prognosis. Treatment modalities include observation, systemic chemotherapy, intravitreal chemotherapy, plaque radiotherapy, external beam radiotherapy, photodynamic therapy, surgical resection, and enucleation for painful, blind eyes [1].

In our case, one cycle of Cisplatin/Pemetrexed was received; however, it was replaced by Crizotinib chemotherapy since the NGS showed ROS 1. This was discontinued after a few days and replaced with Lorlatinib 100 mg daily due to concerns regarding retinal metastasis. As mentioned above, after 2 months of chemotherapy course, PET scan showed a marked regression in retinal metastasis.

Lorlatinib is a third‐generation tyrosine kinase inhibitor (TKI) specifically designed to target anaplastic lymphoma kinase (ALK) and ROS1 rearrangements in non‐small cell lung cancer (NSCLC). Its efficacy is well established in patients with ALK‐positive NSCLC, including those who have developed resistance to earlier‐generation ALK inhibitors [7].

Based on documentation, there have been only two reports documenting the response of choroidal metastasis to systemic treatment with TKIs. Unlike our case, both patients were initially treated with erlotinib, but eventually disease progression necessitated the switch to osimertinib as second‐line treatment [8].

## Author Contributions

Data Collection, Literature Search, Manuscript Preparation: B.A., Supervision, Writing – original draft, Writing – review and editing. A.E., Writing – original draft, Writing – review and editing. O.O.E., Writing – review and editing. M.E., Writing – review and editing. A.M.K., Writing – review and editing. R.E., Writing – review and editing. All authors read and approved the final manuscript.

## Ethics Statement

The article describes a case report. Therefore, no additional permission from our Ethics Committee was required.The authors declare that written informed consent was obtained for the publication of this manuscript and accompanying images and attest that the form used to obtain consent from the patient complies with the Journal requirements. I attest that the original of the signed form(s) is held by the treating institution.

## Conflicts of Interest

The authors declare no conflicts of interest.

## Data Availability

All data generated or analyzed during this study are included in this article.
